# Influenza Herd-Level Prevalence and Seasonality in Breed-to-Wean Pig Farms in the Midwestern United States

**DOI:** 10.3389/fvets.2017.00167

**Published:** 2017-10-11

**Authors:** Fabian Orlando Chamba Pardo, Ana Alba-Casals, Joel Nerem, Robert B. Morrison, Pedro Puig, Montserrat Torremorell

**Affiliations:** ^1^Veterinary Population Medicine Department, University of Minnesota, St. Paul, MN, United States; ^2^Pipestone Veterinary Services, Pipestone, MN, United States; ^3^Departament de Matemàtiques, Universitat Autònoma de Barcelona, Cerdanyola del Vallès, Barcelona, Spain

**Keywords:** influenza, seasonality, prevalence, genetic diversity, swine, absolute humidity, breed-to-wean pig farm, swine influenza virus

## Abstract

Influenza is a costly disease for pig producers and understanding its epidemiology is critical to control it. In this study, we aimed to estimate the herd-level prevalence and seasonality of influenza in breed-to-wean pig farms, evaluate the correlation between influenza herd-level prevalence and meteorological conditions, and characterize influenza genetic diversity over time. A cohort of 34 breed-to-wean farms with monthly influenza status obtained over a 5-year period in piglets prior to wean was selected. A farm was considered positive in a given month if at least one oral fluid tested influenza positive by reverse transcriptase polymerase chain reaction. Influenza seasonality was assessed combining autoregressive integrated moving average (ARIMA) models with trigonometric functions as covariates. Meteorological conditions were gathered from local land-based weather stations, monthly aggregated and correlated with influenza herd-level prevalence. Influenza herd-level prevalence had a median of 28% with a range from 7 to 57% and followed a cyclical pattern with levels increasing during fall, peaking in both early winter (December) and late spring (May), and decreasing in summer. Influenza herd-level prevalence was correlated with mean outdoor air absolute humidity (AH) and temperature. Influenza genetic diversity was substantial over time with influenza isolates belonging to 10 distinct clades from which H1 delta 1 and H1 gamma 1 were the most common. Twenty-one percent of farms had three different clades co-circulating over time, 18% of farms had two clades, and 41% of farms had one clade. In summary, our study showed that influenza had a cyclical pattern explained in part by air AH and temperature changes over time, and highlighted the importance of active surveillance to identify high-risk periods when strategic control measures for influenza could be implemented.

## Introduction

Influenza A virus (IAV) is an economically significant pathogen in pig populations and it has been associated with increased mortality (1), increased feed conversion, and decreased daily weight gain in finishing pigs (2, 3). Hence, influenza can decrease US pork producer’s profitability and affect business continuity if strains are linked to human health (4). IAV is part of the porcine respiratory disease complex in pigs together with porcine reproductive and respiratory syndrome virus (PRRSV), *Mycoplasma hyopneumoniae*, and porcine circovirus type 2 (PCV2).

Breed-to-wean (BTW) pig farms house adult females and their progeny from birth to weaning, which in the USA happens at approximately 21 days of age. BTW farms play a crucial role in influenza epidemiology because piglets can unnoticeably maintain, diversify, and transmit IAV at weaning when they are moved to grow-finish farms (5–7). There is some evidence that IAV infections in pigs are more common in the colder months (8–10) but, in general, information on herd-level prevalence and seasonal distribution of influenza in BTW farms is limited and it is unclear whether seasonality is observed in piglets, given that piglets are born almost daily and for the most part, they are housed in mechanically controlled environments.

There is evidence that certain meteorological conditions such as air temperature and humidity are associated with IAV survivability and transmissibility (11–17). In pigs, indoor-barn air temperature is usually controlled and drives ventilation rates in mechanically ventilated buildings. In contrast, indoor-barn air humidity is not controlled and it depends on outdoor conditions (18). Therefore, meteorological conditions could potentially impact IAV survival and transmission inside swine facilities, which could affect IAV circulation, detection, and prevalence in pig farms.

Intervention strategies to control influenza often reside in the use of vaccines in sows before farrowing (19–21) with the goal to enhance transfer of passive immunity to piglets, decrease risk of infection, and reduce clinical disease presentation (5, 22–27). However, an additional challenge to control influenza is the genetic diversity of strains circulating in pig populations. Co-circulation of genetically distinct viruses is common in pigs in both grow-finish (28–32) and BTW farms (7, 9) which make vaccination difficult to succeed given the limited cross-reactivity among certain strains (33).

In this study, we sought to (i) assess influenza herd-level prevalence and seasonality over time in BTW farms, (ii) investigate the correlation of influenza herd-level prevalence with outdoor air temperature and humidity, and (iii) characterize the genetic diversity of the influenza viruses detected in BTW farms over time. We hypothesize that influenza levels in BTW farms are cyclical and that seasonality can be correlated with certain meteorological conditions. Understanding herd-level prevalence, seasonality, genetic diversity, and meteorological conditions affecting influenza in BTW farms may help guide the allocation and timing of strategies to control influenza in pigs.

## Materials and Methods

### Ethics Statement

The procedures employed as part of this study were approved by the Institutional Animal Care and Use Committee (IACUC) of the University of Minnesota (Protocol no. 1510-33054A). The participating production system agreed to share influenza testing data with the researchers.

### Influenza Active Surveillance Program

Data obtained for this project originated from a swine production system that had been conducting influenza surveillance since 2011 as part of their herd health management program. Briefly, the swine production system had 60 BTW farms located in three Midwestern States (Minnesota, Iowa, and South Dakota). Samples were collected from a representative sample of piglets prior to wean because piglets have been identified as a key subpopulation able to maintain and spread IAV (5–8). The swine production system aimed to collect four monthly oral fluid samples from each farm. Oral fluids were chosen because of better pen-level sensitivity and easiness of collection by farm personnel (34, 35).

Four oral fluid samples aimed to detect a within-herd influenza prevalence of 10% or higher, assuming 100 and 80% diagnostic test specificity and sensitivity, respectively, and 93% within-farm sensitivity (34–36). However, the number of samples collected in a given sampling event ranged between 1 and 7 and the implications for within-farm sensitivity are discussed in Section “Assessment of the Active Surveillance Program.” Followed procedures were also outlined as part of the United States Department of Agriculture (USDA) Influenza Surveillance Program (37).

Collection of oral fluids was performed as described before (36). Briefly, oral fluids were collected from piglets in four farrowing crates choosing one crate per farrowing room. Crates were conveniently selected by farm personnel and the day prior to sample collection, piglets in the selected crates were trained with a different rope to increase the chances of obtaining an oral fluid sample. A cotton rope was hanged in the farrowing crates within reach of the piglets. After the ropes were saturated with oral fluids, the ropes were squeezed into plastic bags to obtain the oral fluid samples that were then refrigerated and submitted to the diagnostic laboratory for IAV testing.

### IAV Testing, Isolation, and Sequencing

Oral fluid samples were tested by reverse transcriptase polymerase chain reaction (RT-PCR) targeting the matrix gene of IAV (38). RT-PCR testing was conducted at South Dakota State University Veterinary Diagnostic Laboratory. A sample was considered positive if the cycle threshold (ct) value was 38 or lower. Isolation of influenza in Madin–Darby Canine Kidney (MDCK) cells was attempted in up to two samples per submission with the lowest ct values according to the USDA Influenza Surveillance Program guidelines. When isolates were available, sequencing of the hemagglutinin gene was attempted (37).

### Meteorological Data

Meteorological data including outdoor air temperature and relative humidity (RH) was compiled from 14 local land-based stations located near the farms. Hourly meteorological data were gathered from the National Centers for Environmental Information (39), and then data were aggregated monthly to conduct the analysis. Monthly average temperature and RH were calculated by including all available hourly data from all stations each month. Absolute humidity (AH) was calculated using hourly temperature and RH following the equation AH = 2.17 × (*p*_w_/*T*) described by McDevitt et al. (14), where *T* is the air temperature in kelvin (K) and *p*_w_ is the partial pressure of water vapor in pascals. *p*_w_ was calculated using the equation *p*_w_ = RH × *P*^o^, where RH is the air relative humidity and *P*^o^ is the saturation vapor pressure in pascals. Finally, *P*^o^ was calculated as follows: *P*^o^ = exp[(−5,800/*T*) + 1.391 − 0.04868 × *T* + 4.176 × 10^−5^ × *T*^2^ − 1.445 × 10^−8^ × *T*^3^ + 6.546 × *lnT*], where *T* is the air temperature in K and *lnT* is the natural logarithm of *T* (14). AH is reported in grams of water per cubic meter of air (g/m^3^). AH data were also averaged monthly including data from all stations.

### Data Analysis

#### Assessment of the Active Surveillance Program

In order to characterize the sensitivity of the surveillance program, the number of monthly oral fluid samples submitted per farm, and the number of positive and total submissions per farm were analyzed. Moreover, the within-farm sensitivity to detect IAV circulation was assessed for each sampling. The within-farm sensitivity was computed using the actual number of oral fluids in each sampling by assuming (a) a within-farm influenza prevalence of 10%, (b) nine piglets chewed the rope in each crate in 30 min (pool size) (40), and (c) a diagnostic sensitivity and specificity for influenza RT-PCR detection in oral fluids of 80% and 100%, respectively (35). The within-farm sensitivity was computed using the *Sep.pooled* function of the *Rsurveillance* package (41) in *R 3.2.3* statistical software (42). Overall, we estimate that we needed at least three oral fluids per submission in order to achieve a within-farm sensitivity of 87%.

#### Influenza Herd-Level Prevalence and Seasonality Analysis

Only those farms that had at least 30 monthly submissions with estimated within-farm sensitivity of at least 87% were included in the time series analysis. The selected level of within-farm sensitivity was chosen in order to achieve a high and uniform level of confidence of the within-farm influenza detection. Influenza monthly herd-level prevalence was estimated by dividing the number of positive farms by the total number of farms submitting in a given month.

Initially, the monthly herd-level prevalence was analyzed using basic statistics and plots. Prevalence was logit transformed to stabilize the variance and normalize the distribution (43). The pattern of influenza herd-level prevalence was evaluated using additive decompositions and classical time series models. Autoregressive integrated moving average (ARIMA) models combined with time as a trend covariate, and trigonometric functions such as sine and cosines (Fourier functions) at different time intervals as covariates were used to assess influenza trend and seasonality. ARIMA models *per se* assume no trend and seasonality (stationary series). However, the combined models allowed us to account and test for trend and seasonality of the influenza herd-level prevalence.

All tested models were fitted using the logit transformed data (44, 45). Several combinations of the trend and trigonometric functions were tested at 3-, 4-, 6-, 12-, and 24-month intervals as linear regression models. Trend, sine, and cosine functions that were significant (*p* < 0.05) were kept for the combined models. Several combined models were tested with different ARIMA models. Model selection was based on parsimony using the lowest Bayesian Information criterion (BIC) as a goodness-of-fit criteria, including residual inspection to ensure lack of autocorrelation through autocorrelation function (ACF) and partial autocorrelation function (PACF) plots. Error distribution plots were checked for normality. These analyses were computed using the *R base* package and the *forecast* package (46) in *R 3.2.3* statistical software (42).

The final combined model was an ARIMA (0, 0, 3) plus sine and cosine functions for 6- and 12-month periods (annual and semiannual cycles). The final model after the logit transformation can be expressed as follows:

$$Y_{t}=\mu +\alpha_{1}\epsilon_{t-\text{1}}+\alpha_{2}\epsilon_{t-2}+\alpha_{3}\epsilon_{t-3}+\beta_{1}\text{sin}(\omega_{1}t)+\beta_{2}\text{cos}(\omega_{1}t)+\beta_{3}\text{sin}(\omega_{2}t)+\beta_{4}\text{cos}(\omega_{2}t)+\epsilon_{t},$$

where *Y_t_* is the logit transformed monthly herd-level prevalence, μ is the intercept, α_i_ is the coefficients of the moving average, *ϵ_t_*_−_ *_i_* is the error terms of the ARIMA model, and β*_i_* is the coefficients of the trigonometric functions: $\omega_{i}=\frac{2\pi }{T_{i}}$, where *T_i_* is the number of months (period) for which seasonality was tested and *ϵ_t_* is the error term of the trigonometric functions model.

#### Meteorological Data and Influenza Herd-Level Prevalence Correlation

To explore the correlation between monthly herd-level prevalence and meteorological conditions, specifically ambient air temperature, RH, and AH, we used the cross-correlation function (*ccf*) in the *R base* package and the *lag2.plot* function in the *astsa* package (47) at different time lags. Lag correlation coefficient *p*-values were obtained using the *pnorm* function of the *R base* package. The above-described statistical analysis was done using *R 3.2.3* statistical software (42). Although at first it may appear redundant to link both temperature and AH to prevalence, AH is a function of both temperature and RH which does not always have to fluctuate in the same direction as temperature.

#### Influenza Genetic Diversity Analysis

Hemagglutinin gene (HA) sequences were annotated for completeness and functionality using the influenza virus sequence annotation tool (FLAN)^1^ (48). H1 clade classification was done using the swine H1 clade classification tool in the Influenza Research Database (IRD)^2^ (49).

For H3 cluster classification, reference strains were obtained from the Influenza Virus Resource at the National Center for Biotechnology Information (NCBI)^3^ (50). Included H3 reference strains were under the following GenBank reference numbers: JX092535 (H3 IV C), JX092307 (H3 IV D), JN652493 (H3 IV F), JN638733 (H3 IV A), JF812276 (H3 IV E), CY114592 (H3 IV B), CY095675 (H3 I), CY006475 (H3 III), CY002120 (H3 II), and KC306165 (H3 humSea11).

Alignment of 1,698-nucleotide-length HA sequences was done using the ClustalW algorithm. Phylogenetic trees were generated using a neighbor joining method and a Hasegawa, Kishino, and Yano (HKY) substitution model in *Geneious version 8.1.8*^4^ (51). H3 cluster was inferred according to the clustering of each isolate in the corresponding trees.

## Results

### Influenza Active Surveillance Program Assessment

From July 2011 to April 2016, there were 58 months of data and 60 BTW pig farms that submitted samples as part of the company influenza surveillance program. The median size of the selected farms was 3,125 sows ranging from 1,200 to 6,000. There were 2,105 diagnostic submissions with a total of 7,778 oral fluids. The number of oral fluids per submission varied from 1 to 7 and the estimated within-farm sensitivity varied from 49 to 100%. Lastly, a total of 34 farms that had both at least 30 submissions and a within-farm sensitivity of 87% or higher (three or more oral fluids per submission) were selected and included in the seasonality analysis.

From the 34 selected farms, there were 1,523 submissions with a total of 6,585 oral fluids. The number of oral fluids per submission varied from 3 to 7 with a median of 4 and an interquartile range (IQR) of 0. The median number of submissions per farm was 47 (IQR = 10) with a range from 30 to 55. Median positive submissions per farm were 12 (IQR = 8) ranging from 3 to 34. Moreover, there were 28% (424/1,523) of submissions with at least one influenza positive result. All farms tested positive at least once during the study period. Finally, the within-farm sensitivity had a median of 93% (IQR = 0) and varied from 87 to 99%.

### Seasonality of Influenza Herd-Level Prevalence

**Figure 1:**
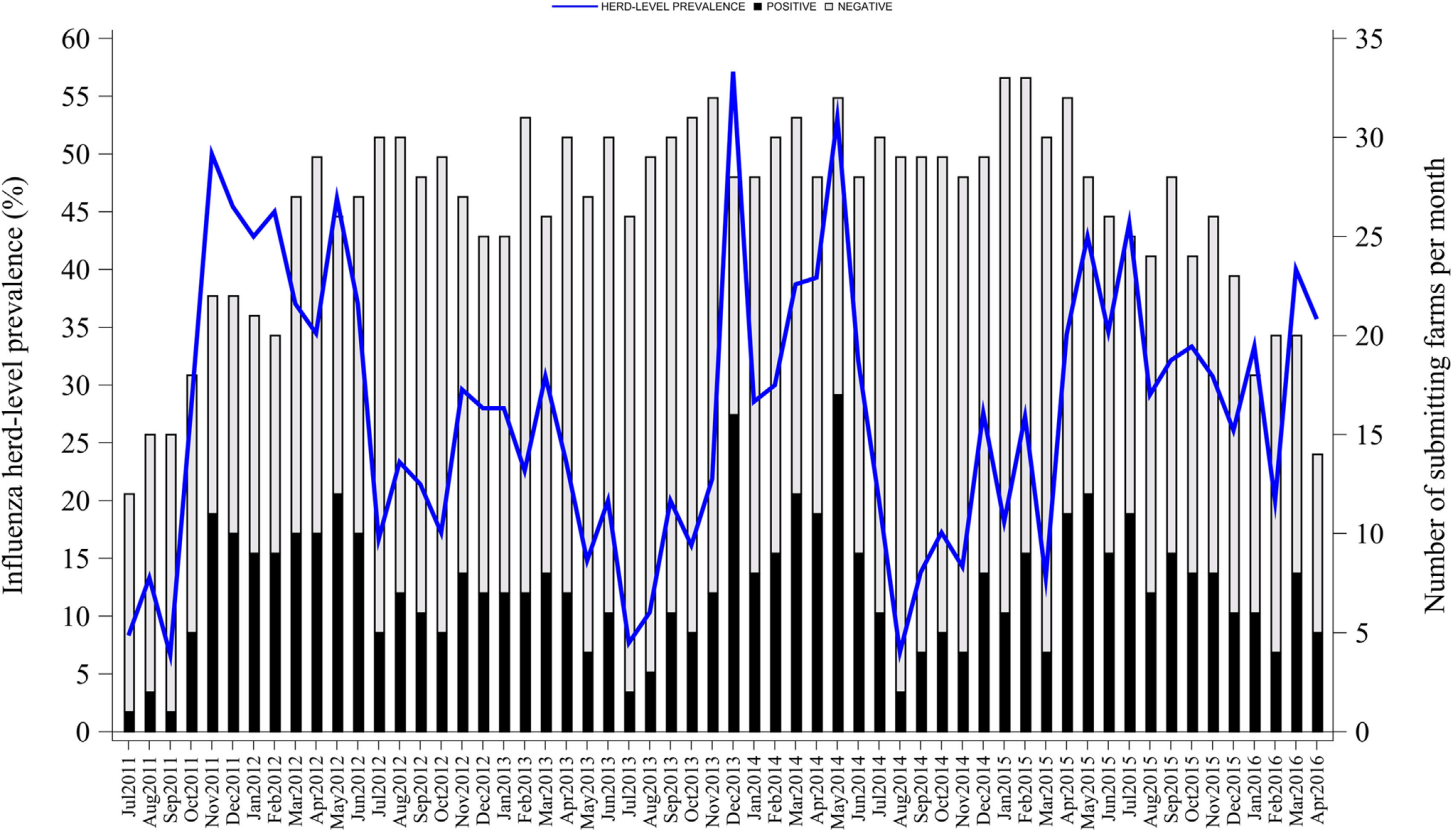
Influenza A virus herd-level prevalence in breed-to-wean pig farms.

**Figure 2:**
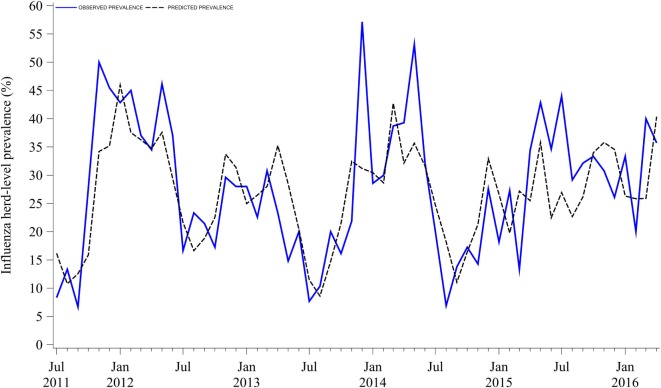
Observed and predicted seasonality of influenza A virus herd-level prevalence in breed-to-wean pig farms.

Influenza herd-level prevalence varied over time. Prevalence of influenza had a median of 28% (IQR = 18%) with a range from 7 to 57%, as shown in Figure 1. Influenza herd-level prevalence showed a seasonal pattern that was repeated on a yearly basis. The seasonality analysis showed that the herd-level prevalence had annual and semiannual cycles that were detected with the sine and cosine functions at 6- and 12-month periods in the final combined model as described above (*p* < 0.05). Prevalence was low in summer, rose during fall, and peaked twice in both early winter (December) and late spring (May). August was the month with the lowest prevalence. Figure 2 shows the seasonality of influenza herd-level prevalence as observed and as predicted after considering the combined final model that included the significant seasonal functions as predictors.

### Meteorological Conditions and Influenza Herd-Level Prevalence

**Figure 3:**
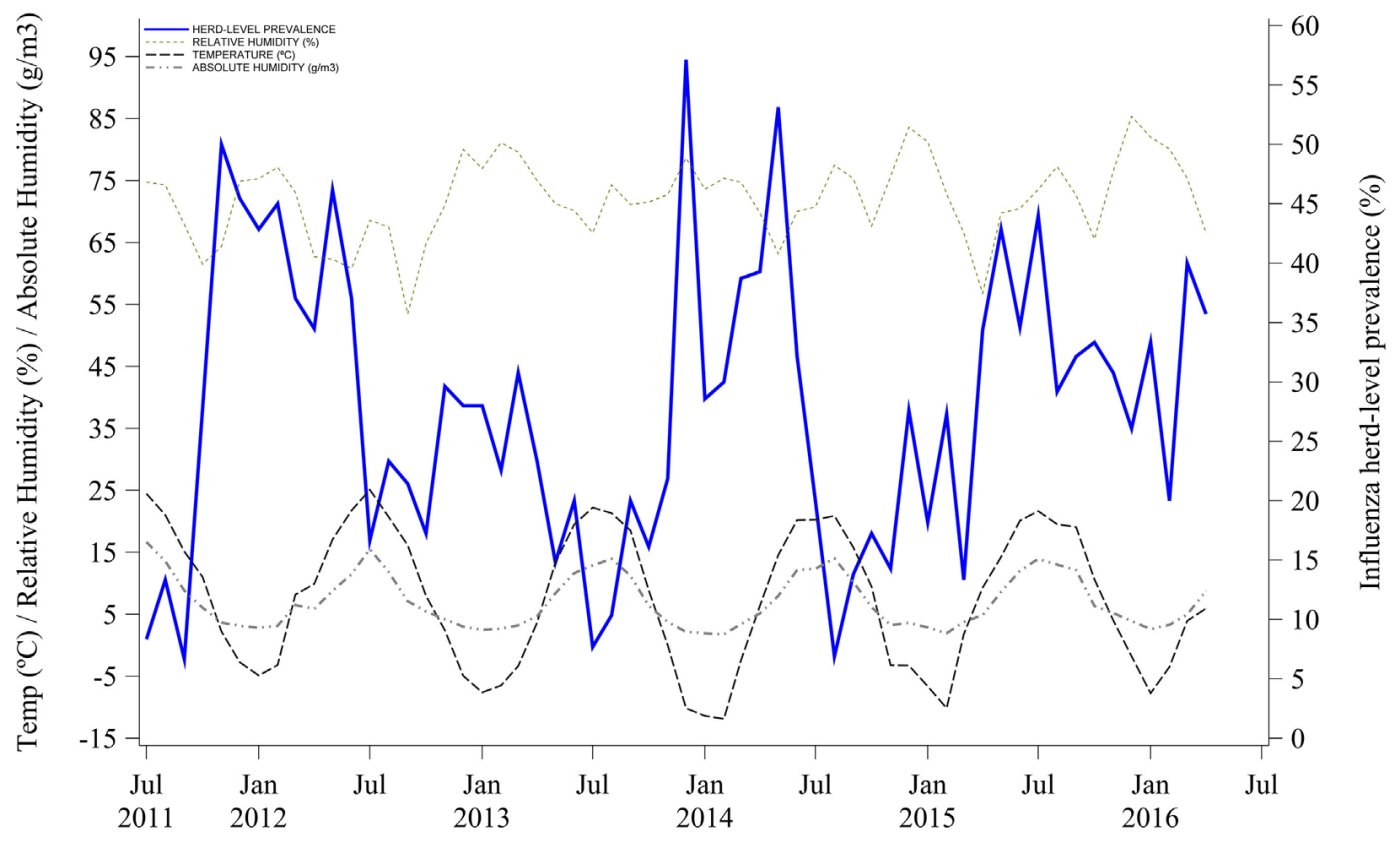
Correlation of meteorological conditions and influenza A virus herd-level prevalence.

Mean outdoor air temperature and AH were negatively correlated with herd-level prevalence. Influenza herd-level prevalence was higher when both mean outdoor air temperature and AH were lower. Indeed, temperature and AH variations that happened earlier at 0, −1, and −2 months were statistically correlated with the influenza herd-level prevalence of a given month, as shown in Table 1 and Figure 3. The lag correlation coefficients varied between −0.28 and −0.43 for both temperature and AH at lags from 0 to −3 months. Lastly, mean outdoor air RH was not correlated with influenza herd-level prevalence at the tested lag months, as shown in Table 1 and Figure 3.

**Table 1:** Lag correlation between influenza A virus herd-level prevalence and meteorological conditions.

Outdoor air conditions	Lag time in months	Lag correlation coefficients	Lag correlation *p*-value
Temperature (°C)	0	−0.28	0.033
	−1	−0.33	0.012
	−2	−0.34	0.010
	−3	−0.24	0.068
Relative humidity (%)	0	−0.08	0.542
	−1	−0.10	0.446
	−2	−0.09	0.493
	−3	−0.02	0.879
Absolute humidity (g/m^3^)	0	−0.35	0.008
	−1	−0.43	0.001
	−2	−0.41	0.002
	−3	−0.21	0.110

### Influenza Co-Circulation and Genetic Diversity at the System Level

Eighty-five (20%) influenza isolates from 424 positive submissions were obtained from the 34 selected farms. Isolates were distinct genetically and grouped into 10 different clades or clusters of H1 and H3 subtypes that are contemporary in North American swine populations. The most common clades identified were H1 delta 1 (40%, 34/85), H1 gamma 1 (21%, 18/85), and clusters H3 IV A (12%, 10/85) and H3 IV B (11%, 9/85).

**Figure 4:**
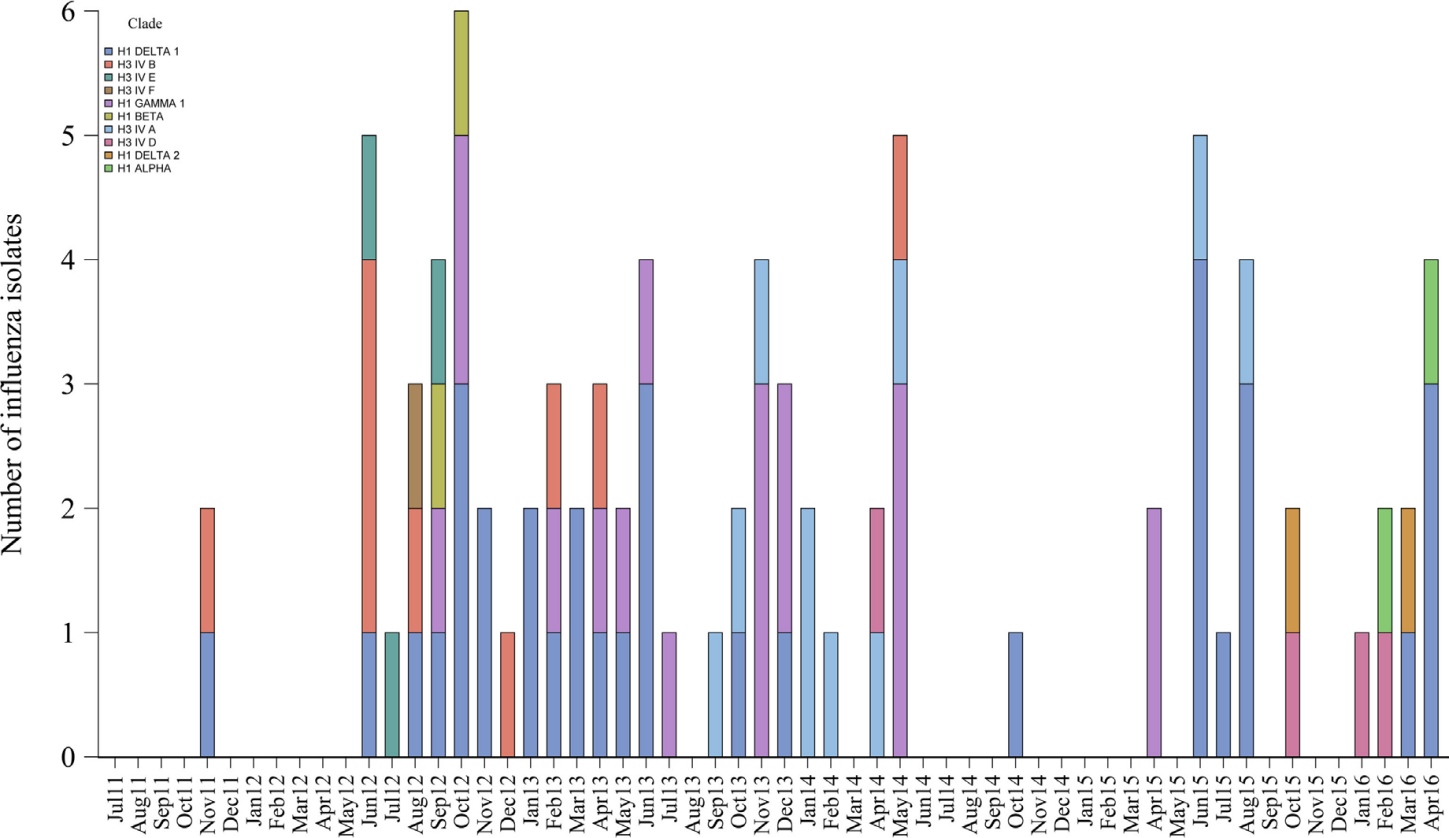
Genetic classification and frequency of influenza A virus isolates over time.

Furthermore, 21% (7/34) of the farms had 3 different influenza genetic clades circulating during the study period, 18% (6/34) had 2, 41% (14/34) had 1, and the remaining 20% (7/34) of farms had no isolates available to further characterize influenza. The genetic diversity and frequency of the influenza isolates obtained over time are presented in Figure 4.

## Discussion

Understanding the temporal dynamics and genetic diversity of influenza in BTW farms is central to control influenza infections in pigs. In this study, we assessed influenza herd-level prevalence, seasonality, and strain distribution in Midwestern BTW farms belonging to one production system. The farms sampled were considered representative of the region, given the similarities across commercial farms in the Midwest and the large number of farms managed by the production company in the study. Influenza infections were widespread and seasonal with infection peaks in both winter and spring. The seasonal pattern was partially explained by air temperature and AH, and there was significant co-circulation of genetically distinct strains over time. To the knowledge of the authors, this is the first time that influenza herd-level prevalence and seasonality in swine has been assessed based on an active surveillance program.

Influenza seasonality in BTW farms is epidemiologically significant because BTW farms supply pigs at weaning. Weaned pigs are commonly transported to distant locations, which represents a risk for influenza dissemination to grow-finish farms (6, 9, 52). Our results targeting the sampling of piglets showed the highest herd-level prevalence in December and May, which is slightly different and after the peaks in October/November and April/May reported in other studies based on the number of positive diagnostic laboratory submissions (53–56) and short-time investigations (8–10, 28). Differences on the age of pigs sampled, geographical regions, climatic conditions, immune status, management practices, sampling strategies, introduction of new strains by replacement females, and/or farm personnel and lack of precision in model predictions used in the studies may explain the differences among the reported studies (57–59).

It is also important to note that our study was based on an active surveillance program with a standardized sampling procedure and high within-farm sensitivity for influenza detection across the participating farms. However, the assumption that nine pigs (pool size) contributed to one oral fluid sample may have overestimated the within-farm sensitivity for a within-farm prevalence of 10% or higher. Given that there are no data available on how many suckling piglets will chew a rope in 30 min, and the fact that piglets were trained to chew the rope before collection (36), we feel that the assumptions to calculate the within-farm sensitivity are logical. Within-farm sensitivity estimates using a pool size of 7 and 8 would still be 80% or higher to detect a within-farm prevalence of 10% or higher and considering three or more oral fluid samples (results not shown). Finally, a BTW farm that tested negative means that within-farm prevalence was lower than 10% considering our sampling approach.

Although the seasonal pattern was repeatable across the 5 years of study, herd-level prevalence varied between years perhaps because of differences in herd immunity, management strategies, or introduction of novel strains through female replacements and/or farm personnel. Analysis of the effect of these factors on herd-level prevalence was beyond the scope of this study. Nevertheless, the consistent seasonal pattern described in these farms can serve as a guide for producers and veterinarians to allocate control strategies to mitigate the spread of influenza.

Influenza seasonality in people and birds has been attributed to seasonal variations in temperature and humidity (12, 15, 60–65). Both temperature and AH have been associated with influenza survivability and transmissibility (11, 14–18, 66–69). In our study, we observed an association between influenza herd-level prevalence and outdoor air temperature and AH. Although the correlation was considered low, seasonal changes in air AH and temperature appeared to partially explain influenza circulation in the studied farms. The mechanism behind this correlation is unclear, given that pigs are housed indoor in mechanically controlled environments. However, given that ventilation controls are set based on ambient temperature, we speculate that outdoor conditions can have an effect on influenza circulation inside facilities. Indeed, ventilation rates may act as a surrogate for outdoor environmental conditions but unfortunately, we had no data on ventilation rates to evaluate whether there was a direct association between ventilation rates and influenza herd-level prevalence. Further studies should investigate this potential association.

The genetic diversity and frequency of influenza isolates was significant despite the low yield of virus isolates obtained from oral fluids, which is known to be a poor sample to measure viral viability (34–36, 70–72). The isolates clustered in 10 genetically distinct hemagglutinin clades or clusters that were contemporary and similar to the ones reported by the USDA influenza surveillance program (53, 56, 73–76). In our study, we could not establish an association between the seasonal peak of infection and the detection of new strains likely because of the limited number of isolates recovered from each farm. This is a question that remains to be answered in future studies.

Furthermore, there were multiple farms that had more than one genetically distinct influenza virus. The co-circulation of different influenza strains in swine farms has been documented before (7, 9, 28), and our results support those studies and illustrate the difficulty to control influenza in BTW farms. The detected co-circulation is relevant because it suggests that comprehensive vaccination approaches that consider the broad genetic diversity are needed to control influenza in swine populations (75).

In summary, influenza herd-level prevalence in Midwestern BTW pig farms had a seasonal pattern with higher levels of influenza in winter and spring months. Seasonality was partially explained by air temperature and AH, although other factors may have played a role in the observed trends. Finally, our results evidenced the co-circulation of genetically diverse influenza viruses over time and highlighted the challenge that this represents for the control of influenza in pigs.

## Author Contributions

FCP gathered data for the study, conducted data analysis, and prepared the manuscript. JN coordinated sample submission and testing. AA-C and PP contributed to the statistical analysis. RM contributed to the results interpretation and manuscript preparation. MT conceived and supervised the study and reviewed, edited, and approved the manuscript.

## Conflict of Interest Statement

The authors declare that the research was conducted in the absence of any commercial or financial relationships that could be construed as a potential conflict of interest.

## Footnotes

^1^https://www.ncbi.nlm.nih.gov/genomes/FLU/Database/annotation.cgi.

^2^http://www.fludb.org.

^3^https://www.ncbi.nlm.nih.gov/genome/viruses/variation/flu/.

^4^http://www.geneious.com.
